# One‐year usage patterns of SGLT‐2 inhibitors and GLP‐1 receptor agonists in individuals with type 2 diabetes in a real‐world population

**DOI:** 10.1111/dom.70269

**Published:** 2025-11-04

**Authors:** Louise A. Donnelly, Katyayeni Singh, Rory J. McCrimmon, Ewan R. Pearson

**Affiliations:** ^1^ Diabetes Endocrinology and Reproductive Biology, School of Medicine University of Dundee Dundee UK

**Keywords:** cohort study, database research, GLP‐1 analogue, real‐world evidence, SGLT2 inhibitor, type 2 diabetes

## Abstract

**Aims:**

Real‐world studies of sodium‐glucose co‐transporter‐2 inhibitors (SGLT2i) and glucagon‐like peptide‐1 receptor agonists (GLP‐1RA) typically assess adherence and discontinuation as separate outcomes. We applied a novel approach that integrates these measures to categorize patterns of drug usage. We describe patterns across individual subclasses, and in overall class‐level models, we examined clinical characteristics associated with each category with particular interest in poor adherence and intolerance.

**Materials and Methods:**

We conducted an observational cohort study using electronic health records from the Scottish Care Information Diabetes Collaboration database, including individuals with type 2 diabetes who initiated a SGLT2i or GLP‐1RA. Each initiation was followed for 1 year and classified into five mutually exclusive groups: adherent, poor adherence and three discontinuation categories including a proxy for intolerance.

**Results:**

Among SGLT2is, poor adherence was more common in younger individuals, with greater socioeconomic deprivation, and higher HbA_1c_, while intolerance was more frequent in older, leaner females and in those with prior genital thrush.

For GLP‐1RAs, newer agents were associated with more favourable usage patterns. Poor adherence was more frequent with liraglutide, lixisenatide, and exenatide compared to semaglutide, and intolerance was more common with lixisenatide. In addition, poor adherence was associated with younger age and prior GLP‐1RA use, while intolerance was linked to lower BMI, female sex, and more advanced CKD.

**Conclusions:**

Distinct clinical and biochemical characteristics are associated with poor adherence versus discontinuation. Understanding these patterns is crucial for developing targeted strategies to improve sustained use, ultimately enhancing treatment outcomes for people with type 2 diabetes.

## 1 INTRODUCTION

Sodium‐glucose co‐transporter‐2 inhibitors (SGLT2i) and glucagon‐like peptide‐1 receptor agonists (GLP‐1RA) are becoming increasingly integral in the management of type 2 diabetes, particularly for individuals with obesity, cardiovascular disease or renal disease risk factors. While clinical trials have demonstrated the numerous benefits of these therapies,^1^, ^2^, ^3^, ^4^, ^5^, ^6^ their full potential may not be realised in real‐world settings due to challenges such as suboptimal adherence and high discontinuation rates. Adverse effects—including genitourinary infections with SGLT2is and gastrointestinal intolerance with GLP‐1RAs—are recognised contributors to early treatment discontinuation and may limit long‐term persistence.^7^, ^8^

Real‐world studies on adherence and discontinuation rates for SGLT2is and GLP‐1RAs have yielded highly variable results, largely due to significant heterogeneity in factors such as healthcare systems, study designs, and outcome definitions.^9^, ^10^ Healthcare systems can be broadly classified into insurance‐based and universal models, with the former often linked to higher rates of poor adherence and discontinuation, primarily driven by cost‐related barriers. Several large studies have evaluated both SGLT2is and GLP‐1RAs within the same cohort, but often focus on the overall class effect, treating medication switching within a class as a continuation of therapy.^11^, ^12^ However, differences in GLP‐1RA subclasses—particularly in administration methods and dosing frequency—have been shown to impact adherence and discontinuation rates.^9^, ^13^ Similarly, while all SGLT2i subclasses share the same mode of administration, variations in adherence and discontinuation rates between subclasses have still been noted.^10^, ^14^

Regarding study outcomes, a ≥80% adherence threshold is commonly used in chronic disease research to define good adherence,^15^, ^16^ however, the definition of discontinuation varies significantly across studies. While a gap of ≥90 days with no medication supply is the most common definition this can range from 60 to 180 days.^11^, ^12^ In a nationwide study of SGLT2is and GLP‐1RAs, where a ≥90‐day gap defined discontinuation, a high proportion of individuals reinitiated therapy within a year. In a sensitivity analysis using a ≥180‐day gap definition, the likelihood of therapy reinitiation remained relatively high.^11^ This suggests that if the study aim is to estimate permanent discontinuation, then using an alternative method, such as a single dispensed prescription, may be necessary.

Adherence and discontinuation are typically studied as separate measures,^17^ yet when combined, they create a spectrum of drug usage, ranging from perfect adherence to near‐immediate, permanent discontinuation (likely due to intolerance). As poor adherence and intolerance stem from different mechanisms, the clinical characteristics associated with each are different. Poor adherence is often influenced by behavioral, cognitive, or psychosocial factors,^18^ whereas intolerance tends to be driven by physiological or pharmacologic factors.^19^ Recognizing these distinctions is essential for delivering effective and sustainable patient care.

In Scotland, with its universal healthcare system providing free prescriptions and a fully integrated national diabetes management system, we used a novel approach to evaluate each available subclass of SGLT2i and GLP‐1RA for 1 year following initiation. We classified patterns of drug usage by combining adherence and discontinuation measures and analyzed the clinical characteristics, with particular interest in poor adherence and intolerance, which have not been widely studied in this context.

## 2 MATERIALS AND METHODS

### 2.1 Data sources

We conducted an observational, population‐based cohort study using electronic health records of individuals with type 2 diabetes in Tayside and Fife, Scotland. Data were collated by the Health Informatics Centre, University of Dundee, with record linkage enabled through the Community Health Index, a unique identifier used across all NHS activity for over 30 years.

The Scottish Care Information—Diabetes Collaboration (SCI‐Diabetes) database provided information on diabetes type, diagnosis date, and BMI. Age, sex, and social deprivation (based on postcode and ranked by quintiles) were obtained from the demography database. Ethnicity data were not collected; as 87% of the Scottish population is white (Scotland's Census 2022), ethnicity was not considered relevant to this analysis. HbA_1c_ and eGFR measurements were obtained from the biochemistry database, and hospital admissions from the ISD SMR01 dataset. Medication data were drawn from the community prescribing database, which captures all encashed prescriptions in Tayside and Fife.

### 2.2 Study population

Eligible individuals had a clinical diagnosis of type 2 diabetes (diagnosed at age ≥35 years) and initiated GLP‐1RA or SGLT2i therapy between 1 January 2008 and 30 April 2022. Prescribing followed national clinical guidelines. While eligibility criteria applied (e.g., eGFR thresholds for SGLT2is and BMI or glycaemic criteria for GLP‐1RAs), there were no formal restrictions preventing prescription for eligible individuals. Follow‐up continued until the earliest of death, leaving the health board area, or 30 April 2023 (end of data availability).

### 2.3 Study design

We identified all initiations of individual subclasses within GLP‐1RAs and SGLT2is, allowing individuals to contribute to more than one subclass. Each initiation was followed for 1 year, with the following exclusions:

1. Less than 1‐year available follow‐up.
2. A prescription for a different subclass within the same drug class (GLP‐1RA or SGLT2i) issued during follow‐up.
3. Initiation of canagliflozin or oral semaglutide, due to low numbers.
4. Initiation of Saxenda (liraglutide), primarily indicated for obesity rather than type 2 diabetes.

A table outlining the derivation of the study is presented in ESM Table 1.

**TABLE 1:** Baseline characteristics of SGLT2i and GLP‐1RA initiations.

Characteristic	SGLT2i	GLP‐1RA
*N*	9091	4637
Age (years)	62.1 (55.2 to 69.1)	60.5 (54.0 to 67.0)
Sex		
Females	3606 (39.7)	2150 (46.4)
Social deprivation		
1 (Most)	1968 (21.6)	1113 (24.0)
2	1892 (20.8)	976 (21.0)
3	1847 (20.3)	931 (20.1)
4	1542 (17.0)	756 (16.3)
5 (Least)	1385 (15.2)	658 (14.2)
(Missing)	457 (5.0)	203 (4.4)
Diabetes duration (years)	8.6 (4.7 to 13.0)	9.8 (6.0 to 14.0)
BMI (kg/m^2^)	33.5 (29.5 to 38.1)	36.7 (33.0 to 41.4)
HbA_1c_ (mmol/mol)	76.0 (67.0 to 87.0)	80.0 (70.0 to 92.0)
eGFR (mL/min/1.73 m^2^)	92.5 (79.3 to 102.1)	92.4 (75.0 to 102.5)
Comedications		
Metformin	7318 (80.5)	3818 (82.3)
Sulphonylureas	3362 (37.0)	2365 (51.0)
TZD	676 (7.4)	852 (18.4)
DPP4‐i	2733 (30.1)	1531 (33)
GLP‐1RA	791 (8.7)	591 (12.7)
SGLT2i	123 (1.4)	808 (17.4)
Insulin	1056 (11.6)	1160 (25.0)
Antihypertensive	6036 (66.4)	3382 (72.9)
Statins	6633 (73.0)	3606 (77.8)
Prior antifungal use		
Yes	1400 (15.4)	911 (19.6)
Subclass		
Exenatide		466 (10.0)
Liraglutide		1987 (42.9)
Exenatide SR		511 (11.0)
Lixisenatide		210 (4.5)
Dulaglutide		899 (19.4)
Semaglutide		564 (12.2)
Dapagliflozin	1772 (19.5)	
Empagliflozin	7319 (80.5)	
Prior subclass count		
0	8714 (95.9)	3505 (75.6)
1	377 (4.1)	927 (20.0)
2		176 (3.8)
>2		29 (0.6)
Year		
2008–2012		1457 (31.4)
2013–2015	694 (7.6)	934 (20.1)
2016–2018	3906 (43.0)	896 (19.3)
2019–2022	4491 (49.4)	1350 (29.1)

*Note*: Data are *N* (%) or Median (IQR).

### 2.4 Defining categories of drug usage

Three metrics were calculated over the 1‐year follow‐up period: (1) proportion of days covered (PDC), defined as the percentage of days with available drug supply; (2) the maximum number of consecutive days without supply (maximum gap); and (3) the total number of prescriptions dispensed. For overlapping prescriptions, remaining supply was carried forward, and hospitalization periods were assumed to be covered.

Five mutually exclusive drug usage categories were defined:

1. Adherent: PDC ≥80%
2. Poor adherence: PDC <80% and maximum gap <90 days
3. Discontinued—standard: Maximum gap ≥90 and <180 days
4. Discontinued—upper bound: Maximum gap ≥180 days with more than one prescription
5. Discontinued—presumed intolerance: Defined as a single dispensed prescription. For clarity and readability, this category is referred to as “intolerance” throughout the manuscript, although we acknowledge that true intolerance cannot be confirmed from dispensing data.

### 2.5 Study outcomes

Primary outcomes were:

1. Proportion of individuals in each drug usage category, stratified by drug subclass (no direct comparisons were made between subclasses due to overlapping individuals)
2. Clinical characteristics associated with each drug usage category. To enable statistical comparisons, subclasses were pooled within each drug class, and one initiation per individual was retained—selecting the most recent to better reflect current clinical practice. Comparisons across drug usage categories focused particularly on differences between the adherent group and those with poor adherence or intolerance.

Although the study used a fixed 1‐year observation period and was not designed to evaluate reinitiation or clinical outcomes, sensitivity analyses were conducted within the empagliflozin subgroup due to its larger sample size. For each drug usage category, we summarised the PDC, maximum gap, and number of prescriptions. Where data were sufficient, further outcomes were assessed:

1. Reinitiation: defined as a prescription for empagliflozin in the year following the initial study period
2. Glycaemic change: defined as the difference in HbA_1c_ between baseline and 1 year
3. BMI change: defined as the difference in BMI between baseline and 1 year

### 2.6 Covariates

Covariates were derived at the time of drug initiation. These included age, sex, social deprivation, duration of diabetes, calendar year of initiation, and prior subclass count (the number of previously initiated subclasses, including those excluded from analysis).

Clinical measures included HbA_1c_ (closest measure within 6 months prior to 7 days post‐initiation), BMI and eGFR (closest measure within 1 year prior to 7 days post‐initiation). CKD stage was defined as: Stage 1 (eGFR ≥90 mL/min/1.73 m^2^), Stage 2 (≥60 and <90), and Stage 3+ (<60).

Comedications (defined as a prescription within 120 days prior to initiation) were grouped as: metformin, sulfonylureas, thiazolidinediones (TZD), DPP‐4 inhibitors (DPP4‐i), insulin, GLP‐1RA, SGLT2i, and statins. Antihypertensives were combined into a single category, including ACE inhibitors, angiotensin receptor blockers, calcium channel blockers, thiazide diuretics, beta‐blockers, and aldosterone antagonists. For the SGLT2i analysis, prior antifungal use was defined as a prescription for fluconazole, clotrimazole, or miconazole within the year prior to initiation.

### 2.7 Statistical Analysis

Descriptive statistics are presented as proportions for categorical variables and as means (standard deviations) or medians (interquartile range) for continuous variables. Associations between clinical characteristics and poor adherence or intolerance (vs. adherence) were assessed using univariable and multivariable logistic regression.

A consistent set of covariates was used across all models to enable comparability. These were limited to variables routinely recorded in clinical practice and selected for demographic or clinical relevance. Model simplicity was prioritized to support interpretability, and potential collinearity was considered.

Covariates included in the models were age (<55, 55 to <60, 60 to <70, ≥70 years), sex, social deprivation (1 = most deprived to 5 = least deprived), BMI (<30, 30 to <35, 35 to <40, ≥40 kg/m^2^), HbA_1c_ (<70, 70 to <80, 80 to <90, ≥90 mmol/mol), CKD stage (1, 2, >2), subclass (empagliflozin and dapagliflozin for SGLT2i models; semaglutide, dulaglutide, liraglutide, exenatide SR, exenatide, and lixisenatide for GLP‐1RA models), and prior subclass (yes/no). In the SGLT2i models prior antifungal use (yes/no) was included as an additional covariate. An additional ‘missing’ group was created for BMI, HbA1c, social deprivation and CKD stage to avoid excluding individuals with missing values from the multivariate model.

For sensitivity analyses of HbA_1c_ and BMI change among empagliflozin users, the measurement closest to 1 year post‐initiation (within a 3–18‐month window) was used. Change was calculated as the difference between baseline and follow‐up. Multiple linear regression models were used to assess differences across drug usage categories, adjusted for the baseline measure.

All analyses were conducted using R (version 4.3.1). Figures were generated using Python. A *p*‐value <0.05 was considered statistically significant.

## 3 RESULTS

### 3.1 Baseline Characteristics

The final study population included 9091 SGLT2i initiations from 8815 individuals and 4637 GLP‐1RA initiations from 3885 individuals. Baseline characteristics are summarised in Table 1. Compared with GLP‐1RA users, those initiating SGLT2is were less likely to be female (39.7% vs. 46.4%), were older (median age [IQR]: 62.1 [55.2–69.1] vs. 60.5 [54.0–67.0] years), and had lower BMI (33.5 [29.5–38.1] vs. 36.7 [33.0–41.4] kg/m^2^) and HbA_1c_ (76 [67–87] vs. 80 [70–92] mmol/mol).

Among SGLT2i users, 1772 individuals initiated dapagliflozin between 2013 and 2022, and 7319 initiated empagliflozin between 2015 and 2022. Among GLP‐1RA users, 466 initiated exenatide (2008–2013), 1987 liraglutide (2010–2021), 511 exenatide SR (2012–2021), 210 lixisenatide (2013–2016), 899 dulaglutide (2016–2022), and 564 semaglutide (2019–2022).

At baseline, 808 GLP‐1RA users (17%) were concurrently using an SGLT2i, and 791 SGLT2i users (9%) were concurrently using a GLP‐1RA.

### 3.2 Drug Usage Categories by Subclass

**FIGURE 1:**
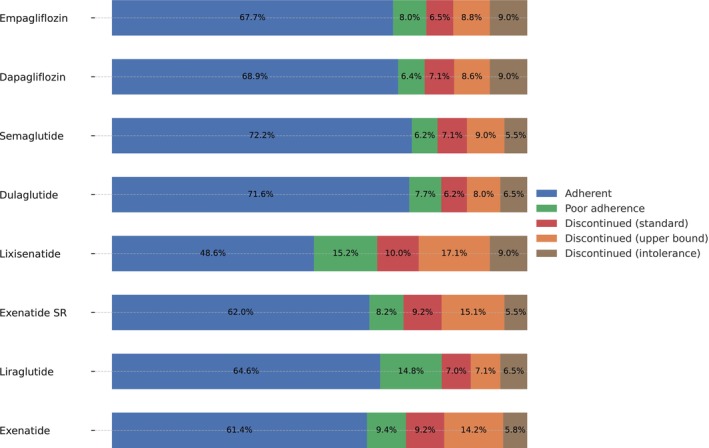
Drug usage categories by GLP‐1RA and SGLT2i subclass. Stacked bar chart showing the proportions of individuals in each category for GLP‐1RA (Exenatide, Liraglutide, Exenatide SR, Lixisenatide, Dulaglutide, Semaglutide) and SGLT2i (Dapagliflozin, Empagliflozin) subclasses. Usage was classified as: Adherent (proportion of days covered (PDC) ≥80%), poor adherence (PDC <80% with no medication gap ≥90 days), and three discontinuation categories: Standard: 90 to 180‐day medication gap, upper bound: ≥180‐day medication gap, and intolerance: only one prescription dispensed.

A breakdown of drug usage categories is shown in Figure 1. Usage patterns were broadly similar across SGLT2i subclasses: one‐year adherence was 69% for dapagliflozin and 68% for empagliflozin; discontinuation rates were 25% and 24%, respectively; and intolerance rates were 9% for both.

In contrast, GLP‐1RA subclasses showed more variability. Adherence was highest for semaglutide and dulaglutide (both 72%). Discontinuation was lowest for dulaglutide, liraglutide, and semaglutide (21–22%) and highest for lixisenatide (36%), exenatide SR (30%), and exenatide (29%). Intolerance rates were generally 5–7%, except for lixisenatide (9%).

### 3.3 Sensitivity analyses for empagliflozin

Among 7319 empagliflozin users, 5955 (81.4%) had at least one additional year of follow‐up (Table 2). The reinitiation rate across all discontinuation groups was 19%, decreasing to 10% for the ‘upper bound’ and intolerant groups, and 2% in the intolerant group alone. In the standard discontinuation group, reinitiation occurred in 45% of individuals, suggesting that discontinuation within this range was not always permanent and that patterns of medication use varied between individuals. In the analyses of glycaemic and BMI change, baseline HbA_1c_ was highest in the poor adherence group, while baseline BMI was lowest in the intolerant group. The adherent group achieved the greatest reductions in both HbA_1c_ and BMI. After adjusting for baseline values, all other groups demonstrated significantly smaller reductions in these outcomes compared to the adherent group, with the discontinuation groups having the poorest outcomes.

**TABLE 2:** Clinical outcome sensitivity analyses in empagliflozin users.

	Adherent	Poor adherence	Discontinued standard	Discontinued upper bound	Discontinued intolerance
Drug usage categorisation metrics					
*N*	4954 (67.7)	585 (8.0)	478 (6.5)	642 (8.8)	660 (9.0)
PDC (%)	98.4 (93.4–100)	74.8 (66.3–76.7)	56.6 (46.3–62.2)	30.7 (23.0 to 38.4)	15.3 (7.7–15.3)
Maximum gap (days)	5 (0–15.0)	60.0 (44–76)	133 (111.2–150.8)	245 (207.2 to 260)	309 (309–337)
Number of prescriptions	7 (7.0–8.0)	5.0 (5.0–6.0)	4.0 (3.0–5.0)	2.0 (2.0–3.0)	1.0 (1.0–1.0)
Reinitiation					
*N* with 2‐year follow‐up	4060	469	390	523	513
Prescription during follow‐up	95.4%	79.3%	44.6%	18.0%	1.9%
Glycaemic change					
*N* with measures	4387	501	421	559	566
Baseline HbA_1c_ (mmol/mol)	77.7 (15.9)	81.1 (17.4)	78.5 (16.0)	78.2 (15.7)	77.6 (15.2)
One year change^a^	−13.6 (15.9)	−11.1 (17.2)	−5.3 (19.5)	−6.1 (18.0)	−5.5 (18.7)
Coefficient (95% CI)^b^	REF	4.5 (3.2–5.8)^c^	8.7 (7.3–10.1)^c^	7.8 (6.6–9.0)^c^	8.0 (6.8–9.2)^c^
BMI change					
*N* with measures	3433	382	334	442	413
Baseline BMI (kg/m^2^)	34.3 (6.6)	34.3 (6.9)	34.4 (7.6)	34.3 (7.2)	33.4 (6.8)
One year change^a^	−1.5 (1.9)	−1.2 (2.1)	−0.9 (2.0)	−0.6 (2.2)	−0.6 (1.9)
Coefficient (95% CI)^b^	REF	0.4 (0.1–0.6)^c^	0.6 (0.4–0.8)^c^	0.9 (0.7–1.1)^c^	0.9 (0.7–1.1)^c^

*Note*: Data are *N* (%) or median (IQR), or mean (SD).

^a^ Unadjusted 1 year change, calculated as measure at 1 year—baseline measure, where a negative value indicates a reduction.

^b^ Values are coefficients (95% CI) from regression models adjusted for baseline, adherent group were the reference category and a higher positive value indicates a smaller reduction in comparison.

^c^ *p* <0.0001 compared with REF.

### 3.4 SGLT2i: Baseline Characteristics by Drug Usage Category

Among 8815 individuals with SGLT2i initiation, baseline characteristics varied by drug usage category (Table S2). The poor adherence group was youngest, most socioeconomically deprived, and had the highest BMI and HbA_1c_. They also had the highest eGFR and shortest diabetes duration, likely reflecting their younger age. In contrast, the intolerant group was oldest and had the lowest BMI and eGFR. Prior antifungal use showed a trend, increasing across groups from adherent to intolerant.

### 3.5 SGLT2i: Clinical Characteristics Associated with Poor Adherence

**FIGURE 2:**
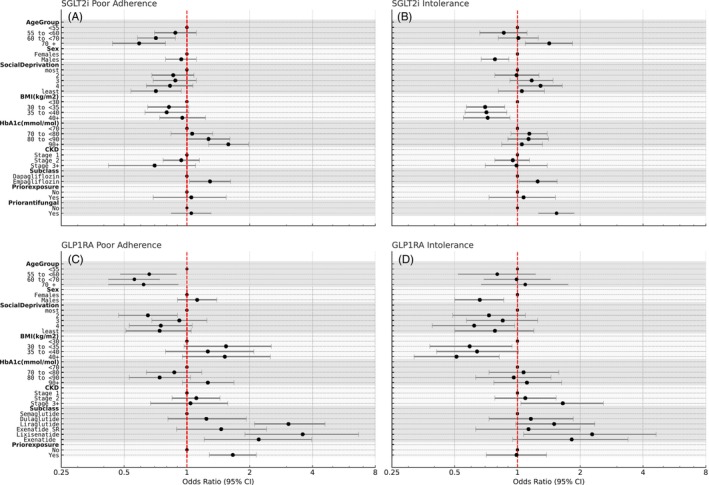
Clinical characteristics associated with poor adherence and intolerance to SGLT2is and GLP‐1RAs. Forest plots showing adjusted odds ratios and 95% confidence intervals from multivariable logistic regression models. Outcomes shown are (A) poor adherence and (B) intolerance to SGLT2is, and (C) poor adherence and (D) intolerance to GLP‐1RAs. Results are presented relative to individuals who were adherent. Reference categories are indicated by an OR of 1.0. All models were adjusted for age, sex, social deprivation, BMI, HbA_1c_, CKD stage, drug subclass, and prior drug exposure. Models for SGLT2is additionally included prior antifungal use.

Full univariable and multivariable models are provided in Table S3. In univariable analysis, poor adherence was associated with younger age, greater social deprivation, higher HbA_1c_, earlier CKD stage, and empagliflozin use (vs. dapagliflozin). In the multivariable model (Figure 2A), all associations remained significant except CKD stage which was no longer significant after adjustment for age.

### 3.6 SGLT2i: Clinical Characteristics Associated with Intolerance

Full univariable and multivariable models are provided in Table S4. In univariable analysis, intolerance was associated with older age, female sex, lower BMI, and prior antifungal use. In the multivariable model, all associations remained significant. Additionally, intolerance was also more likely with empagliflozin than with dapagliflozin (Figure 2B).

### 3.7 GLP‐1RA: Baseline Characteristics by Drug Usage Category

Among 3885 individuals initiating GLP‐1RAs (Table S5), the poor adherence group had the lowest proportion of semaglutide, dulaglutide, exenatide SR, and exenatide users, and the highest proportion of liraglutide users. They were also the youngest and had the highest BMI. The intolerant group had the highest proportion of GLP‐1RA–naïve individuals, and females.

### 3.8 GLP‐1RA: Clinical Characteristics Associated with Poor Adherence

Full univariable and multivariable models are provided in Table S6. In univariable analysis, poor adherence was associated with younger age, greater social deprivation, higher BMI, and use of lixisenatide, liraglutide, or exenatide SR compared to semaglutide. In the multivariable model (Figure 2C), younger age, use of liraglutide, lixisenatide, prior GLP‐1RA exposure, and exenatide (not exenatide SR) were significantly associated with poor adherence.

### 3.9 GLP‐1RA: Clinical Characteristics Associated with Intolerance

Full univariable and multivariable models are provided in Table S7. In univariable analysis, intolerance was associated with female sex, lower BMI, advanced CKD stage, and lixisenatide compared to semaglutide. All associations remained significant in the multivariable model (Figure 2D).

## 4 DISCUSSION

### 4.1 Summary

In this large, population‐based observational study of individuals with type 2 diabetes, we used a novel approach that integrates adherence and discontinuation to categorize patterns of SGLT2i and GLP‐1RA use over a 1‐year period. Among SGLT2i users, poor adherence was more common in younger individuals, those with greater social deprivation, and those with higher baseline HbA_1c_, while intolerance was more frequent in older adults, women, individuals with lower BMI, and those with prior antifungal medication use. For GLP‐1RAs, usage patterns differed across subclasses: poorer adherence was observed with liraglutide, lixisenatide, and exenatide compared with semaglutide, while intolerance was notably higher with lixisenatide. Poor adherence in this group was further associated with prior GLP‐1RA exposure and younger age, whereas intolerance was linked to female sex, lower BMI, and more advanced CKD.

We observed differences in drug usage patterns between SGLT2i subclasses, with empagliflozin showing a stronger association with both poor adherence and intolerance compared to dapagliflozin. This is unexpected, as both drugs are once‐daily oral medications with similar efficacy and safety profiles, and there is no clear pharmacological reason for the differences.^20^ One possible explanation is residual confounding due to changes in prescribing patterns or patient characteristics over time—empagliflozin became the dominant SGLT2i from 2015, and our models used the most recent initiation, which may have introduced bias. Sensitivity analyses using first initiation data showed consistent results, except the difference in intolerance between subclasses disappeared, aligning with the equal proportions in Figure 1. Notably, the association with poor adherence persisted, suggesting it may be more robust. Given the lack of a significant association in the univariable intolerance model, any true difference in tolerability between the two agents is likely small and should be interpreted with caution.

In addition to the SGLT2is, our study highlighted variability within the GLP‐1RA subclasses. Newer formulations, specifically injectable semaglutide and dulaglutide, demonstrated higher adherence compared to older agents. Intolerance rates were similar across most GLP‐1RA subclasses, generally lower than those seen with SGLT2is, except for lixisenatide, which had higher intolerance compared to the adherent group (Figures 1 and 2B). Liraglutide, lixisenatide, and exenatide were more frequently associated with poor adherence compared to semaglutide (Figures 1 and 2A). No significant differences were observed between the weekly administered agents, dulaglutide and exenatide SR. These findings align with literature suggesting that simpler dosing regimens improve adherence.^21^, ^22^, ^23^

Our finding that poor adherence to SGLT2is was more common among younger individuals, those with higher baseline HbA_1c_, and those from more socially deprived backgrounds aligns with previous research.^24^ Social deprivation is a well‐established barrier to adherence, and it is notable that in Scotland, where prescriptions are free, financial cost is unlikely to be the primary driver—suggesting that other structural or behavioral factors may be contributing. The association between higher HbA_1c_ and poor adherence is particularly important, as previous studies have shown that suboptimal adherence is associated with smaller reductions in HbA_1c_.^25^ In our analysis of clinical outcomes among empagliflozin users (Table 2), we found that poor adherence was associated with significantly smaller reductions in HbA_1c_ and BMI compared to the adherent group. These findings highlight that suboptimal adherence, even without full discontinuation, negatively affects clinical outcomes and identifies younger individuals as an important target for interventions and closer follow‐up.

Interpretation of the GLP‐1RA cohort was limited by a smaller sample size, and heterogeneity across subclasses. However, like SGLT2 inhibitors, younger age was again associated with poor adherence, and social deprivation was significant in univariable analysis, though not in the multivariable model. These findings suggest that common demographic patterns may underlie poor adherence across both drug classes, although the strength and consistency of associations may vary.

As hypothesised, clinical characteristics associated with intolerance differed from those linked to poor adherence. For SGLT2is, intolerance was more common among older individuals, females, and those with lower BMI—factors previously linked to increased susceptibility to adverse drug effects.^26^, ^27^ Additionally, individuals who discontinued due to intolerance had a higher prevalence of prior antifungal use, suggesting that a history of genital infections may increase the risk of side effects, a known issue with SGLT2is.^28^

In the GLP‐1RA group, intolerance was more common among females and individuals with lower BMI, although we did not observe an association with older age. Additionally, intolerance was also more prevalent among individuals with more advanced CKD. One possible explanation is increased susceptibility to gastrointestinal side effects in this population, as previously suggested in expert guidance.^29^ However, this mechanism is unlikely to apply uniformly across the class and may be more relevant to specific subclasses, such as exendin‐4‐based GLP‐1RAs that are renally excreted. In our analysis, lixisenatide was more frequently associated with intolerance, supporting the possibility of agent‐specific effects in people with impaired kidney function.

Our definition of intolerance was based on a single prescription encashed within the first year of treatment. While we cannot definitively attribute early discontinuation to adverse effects, several observations support the validity of our definition. SGLT2is are known to cause genitourinary side effects, and individuals in the intolerant group had the highest rates of antifungal prescriptions, suggesting a link between side effects and discontinuation. Furthermore, fewer than 2% of intolerant empagliflozin users (Table 2) encashed a prescription in the following year, indicating likely permanent discontinuation. These findings provide internal validation and suggest that we have identified a clinically meaningful group likely to have discontinued due to treatment‐related factors.

### 4.2 Strengths

A key strength of this study is the use of comprehensive, longitudinal healthcare data, including clinical variables such as BMI, HbA_1c_, and social deprivation. This allows for a robust analysis of real‐world prescribing patterns in a large, representative cohort of individuals with type 2 diabetes. Importantly, our prescribing data, based on actual medication dispensation, provides a more accurate reflection of adherence. Another strength is our PDC calculation, which accounts for hospitalisation periods—a factor often not included in similar studies. This is particularly relevant given the age and comorbidity profile of our population. By including hospital admissions, we avoid underestimating adherence and overestimating discontinuation.

Additionally, our novel approach to drug usage classification differentiates poor adherence, standard discontinuation, and intolerance, while also evaluating multiple subclasses. This allows us to capture a broader spectrum of medication‐taking behaviours and examine variation within these drug classes—an aspect not typically addressed in studies focused on class‐level effects.

### 4.3 Limitations

Our study spanned January 2008 to April 2023, a period during which prescribing patterns for GLP‐1RAs and SGLT2is likely evolved due to updated clinical guidelines and the introduction of newer agents. Consequently, differences observed between dapagliflozin and empagliflozin may partly reflect temporal shifts in clinical practice rather than pharmacological variation. Importantly, the study period concluded before the onset of global GLP‐1RA shortages in June 2023 and does not include oral semaglutide, which has since become more widely used. These factors may limit the generalisability of our findings to the current treatment landscape. Although we did not observe significant changes in adherence or discontinuation during the COVID‐19 pandemic, we noted a drop in initiations followed by a rise from 2021, suggesting temporary disruptions in care that were not fully captured. Adherence metrics derived from dispensing data are proxy measures and may not perfectly reflect actual medication use. Prescription collection does not guarantee that patients took the medication as prescribed, and some misclassification between poor adherence and intolerance may remain despite our efforts to internally validate the intolerance definition. Sensitivity analyses were limited to empagliflozin users due to the small number of individuals initiating GLP‐1RA subclasses or dapagliflozin and should be interpreted as exploratory rather than generalisable to the whole study population. Similarly, the absence of ethnicity data—although reflective of the predominantly White Scottish population—limits extrapolation to more diverse populations. Finally, our study was conducted within a universal healthcare system with free prescriptions; adherence patterns may differ in insurance‐based systems where medication cost and reimbursement policies influence persistence and discontinuation.

## 5 CONCLUSIONS

Our study highlights that poor adherence and discontinuation are driven by distinct clinical and biological factors. Understanding these differences is key to developing targeted strategies that improve adherence, reduce discontinuation, and ultimately enhance patient outcomes.

## CONFLICTS OF INTEREST STATEMENT

The authors declare no conflicts of interest.

## Supporting information


**DATA S1.** Supporting information.

## Data Availability

The data that support the findings of this study are not openly available for reasons of sensitivity but are available from the corresponding author upon reasonable request.
