# Supplementary material for: One‐year usage patterns of SGLT‐2 inhibitors and GLP‐1 receptor agonists in individuals with type 2 diabetes in a real‐world population
Source: Diabetes Obes Metab. 2025 Nov 4;28(2):972–81. doi: 10.1111/dom.70269 (PMC12803620; doi:10.1111/dom.70269)
Supplement: Supplementary file 1 — DATA S1. Supporting information. [file DOM-28-972-s001.docx]

**ESM Table 1Study population derivation**

| **SGLT2-i** | | | **GLP1-RA** | | | | | | |
| --- | --- | --- | --- | --- | --- | --- | --- | --- | --- |
| All* | Dapagliflozin | Empagliflozin | All* | Exenatide | Liraglutide | Exenatide SR | Lixisenatide | Dulaglutide | Semaglutide |
| Total number of individuals before exclusions | | | | | | | | | |
| 13234* | 2921 | 10164 | 7870* | 1189 | 2647 | 777 | 326 | 1347 | 1023 |
| Excluded due to prescription for another subclass within one-year | | | | | | | | | |
| 151* | 71 | 72 | 585* | 144 | 116 | 96 | 76 | 98 | 43 |
| Excluded due to not having one-year available follow up | | | | | | | | | |
| 3992* | 1078 | 2773 | 2648* | 579 | 544 | 170 | 40 | 350 | 416 |
| Final study population | | | | | | | | | |
| 9091 | 1772 | 7319 | 4637 | 466 | 1987 | 511 | 210 | 899 | 564 |

* *These totals include subclasses with small numbers (i.e. canagliflozin, oral semaglutide and Saxenda)*

**ESM Table 2 SGLT2i baseline characteristics by drug usage category**

|  |  | Adherent | Poor adherence | Discontinued Standard | Discontinued  Upper Bound | Discontinued  Intolerance |
| --- | --- | --- | --- | --- | --- | --- |
| N (%) |  | 6052 (68.7) | 680 (7.7) | 575 (6.5) | 747 (8.5) | 761 (8.6) |
| Subclass | Dapagliflozin | 1126 (18.6) | 99 (14.6) | 103 (17.9) | 115 (15.4) | 120 (15.8) |
|  | Empagliflozin | 4926 (81.4) | 581 (85.4) | 472 (82.1) | 632 (84.6) | 641 (84.2) |
| Prior subclass | Yes | 256 (4.2) | 29 (4.3) | 26 (4.5) | 30 (4.0) | 36 (4.7) |
| Age | years | 62.2 (55.5 - 69.1) | 59.6 (53.2- 65.7) | 62.3(55.2 - 69.2) | 62.4 (54.9 - 70.2) | 63.9 (56.5 - 71.3) |
| Sex | Female | 2270 (37.5) | 272 (40.0) | 245 (42.6) | 358 (47.9) | 336 (44.2) |
| Social Deprivation | 1(Most) | 1285 (21.2) | 175 (25.7) | 130 (22.6) | 164 (22.0) | 148 (19.4) |
|  | 2 | 1257 (20.8) | 141 (20.7) | 133 (23.1) | 150 (20.1) | 145 (19.1) |
|  | 3 | 1233 (20.4) | 139 (20.4) | 113 (19.7) | 145 (19.4) | 166 (21.8) |
|  | 4 | 1006 (16.6) | 103 (15.1) | 103 (17.9) | 137 (18.3) | 151 (19.8) |
|  | 5 (Least) | 972 (16.1) | 84 (12.4) | 71 (12.3) | 102 (13.7) | 121 (15.9) |
|  | (Missing) | 299 (4.9) | 38 (5.6) | 25 (4.3) | 49 (6.6) | 30 (3.9) |
| Diabetes duration |  | 8.8 (4.9 - 13.1) | 7.7 (4.2 -12.1) | 8.4 (4.5 - 13.0) | 8.3 (4.4 - 12.7) | 8.6 (5.1 - 13.4) |
| Year | 2013-2015 | 400 (6.6) | 47 (6.9) | 38 (6.6) | 51 (6.8) | 36 (4.7) |
|  | 2016-2018 | 2626 (43.4) | 274 (40.3) | 253 (44.0) | 325 (43.5) | 295 (38.8) |
|  | 2019-2022 | 3026 (50.0) | 359 (52.8) | 284 (49.4) | 371 (49.7) | 430 (56.5) |
| BMI (kg/m²) |  | 33.5 (29.7 - 38.0) | 33.8 (29.4 - 38.7) | 33.4(29.3 - 38.4) | 33.2 (29.1 - 38.0) | 32.6 (28.7 - 37.7) |
| HbA1c (mmol/mol) |  | 76.0 (66.0 - 87.0) | 80.0 (68.0 - 92.0) | 78.0(68.0 - 88.0) | 76.0 (68.0 - 87.0) | 76.0 (67.0 - 88.0) |
| eGFR (mL/min/1.73m²) |  | 92.4 (79.3 - 101.9) | 96.0 (84.3 - 104.7) | 91.8 (78.4 - 102.5) | 91.6 (77.8 - 101.8) | 91.1 (75.2 - 101.2) |
| Comedications: | Metformin | 4995(82.5) | 533(78.4) | 441(76.7) | 577(77.2) | 544(71.5) |
|  | Sulphonylureas | 2266(37.4) | 235(34.6) | 200(34.8) | 316(42.3) | 250(32.9) |
|  | TZD | 445(7.4) | 49(7.2) | 42(7.3) | 69(9.2) | 48(6.3) |
|  | DPP4-i | 1886(31.2) | 192(28.2) | 152(26.4) | 222(29.7) | 191(25.1) |
|  | GLP-1RA | 551(9.1) | 49(7.2) | 47(8.2) | 55(7.4) | 51(6.7) |
|  | SGLT2i | 92 (1.5) | 8 (1.2) | 8 (1.4) | 5 (0.7) | 10 (1.3) |
|  | Insulin | 719 (11.9) | 69 (10.1) | 73 (12.7) | 63 (8.4) | 87 (11.4) |
|  | Antihypertensives | 4154(68.6) | 406(59.7) | 348(60.5) | 481(64.4) | 466(61.2) |
|  | Statins | 4576(75.6) | 473(69.6) | 371(64.5) | 525(70.3) | 480(63.1) |
| Prior antifungal use | Yes | 845 (14.0) | 109 (16.0) | 104 (18.1) | 140 (18.7) | 153 (20.1) |

**ESM Table 3 Clinical characteristics associated with poor adherence versus adherence among SGLT2i users**

|  |  | Adherent | Poor adherence | OR (univariable) | OR (multivariable) |
| --- | --- | --- | --- | --- | --- |
| Age group | <55 | 1417 (86.9) | 214 (13.1) | ref | ref |
|  | 55 to <60 | 1057 (88.5) | 138 (11.5) | 0.86 (0.69-1.08, p=0.211) | 0.88 (0.70-1.11, p=0.281) |
|  | 60 to <70 | 2253 (90.9) | 225 (9.1) | **0.66 (0.54-0.81, p<0.001)** | **0.71 (0.58-0.88, p=0.001)** |
|  | ≥70 | 1325 (92.8) | 103 (7.2) | **0.51 (0.40-0.66, p<0.001)** | **0.59 (0.44-0.79, p<0.001)** |
| Sex | Female | 2270 (89.3) | 272 (10.7) | ref | ref |
|  | Male | 3782 (90.3) | 408 (9.7) | 0.90 (0.77-1.06, p=0.204) | 0.94 (0.79-1.11, p=0.455) |
| Social Deprivation | 1(Most) | 1285 (88.0) | 175 (12.0) | ref | ref |
|  | 2 | 1257 (89.9) | 141 (10.1) | 0.82 (0.65-1.04, p=0.106) | 0.86 (0.68-1.08, p=0.200) |
|  | 3 | 1233 (89.9) | 139 (10.1) | 0.83 (0.65-1.05, p=0.116) | 0.88 (0.69-1.11, p=0.277) |
|  | 4 | 1006 (90.7) | 103 (9.3) | **0.75 (0.58-0.97, p=0.030)** | 0.83 (0.64-1.07, p=0.159) |
|  | 5(Least) | 972 (92.0) | 84 (8.0) | **0.63 (0.48-0.83, p=0.001)** | **0.71 (0.54-0.94, p=0.017)** |
|  | (Missing) | 299 (88.7) | 38 (11.3) | 0.93 (0.63-1.34, p=0.716) | 0.90 (0.61-1.30, p=0.601) |
| BMI (kg/m^2^) | <30 | 1427 (89.5) | 167 (10.5) | ref | ref |
|  | 30 to <35 | 1751 (90.9) | 176 (9.1) | 0.86 (0.69-1.07, p=0.181) | 0.82 (0.65-1.02, p=0.076) |
|  | 35 to <40 | 1273 (90.5) | 133 (9.5) | 0.89 (0.70-1.13, p=0.354) | 0.80 (0.63-1.02, p=0.074) |
|  | ≥40 | 952 (88.2) | 127 (11.8) | 1.14 (0.89-1.46, p=0.295) | 0.95 (0.74-1.23, p=0.720) |
|  | missing | 649 (89.4) | 77 (10.6) | 1.01 (0.76-1.34, p=0.925) | 0.91 (0.68-1.22, p=0.547) |
| HbA_1c_ (mmol/mol) | <70 | 2072 (91.6) | 191 (8.4) | ref | ref |
|  | 70 to <80 | 1434 (91.0) | 142 (9.0) | 1.07 (0.85-1.35, p=0.537) | 1.06 (0.84-1.33, p=0.635) |
|  | 80 to <90 | 1070 (89.0) | 132 (11.0) | **1.34 (1.06-1.69, p=0.015)** | **1.27 (1.00-1.61, p=0.046)** |
|  | ≥90 | 1073 (86.5) | 168 (13.5) | **1.70 (1.36-2.12, p<0.001)** | **1.58 (1.27-1.98, p<0.001)** |
|  | missing | 403 (89.6) | 47 (10.4) | 1.27 (0.89-1.76, p=0.171) | 1.23 (0.86-1.73, p=0.238) |
| CKD stage | 1 | 3215 (88.7) | 409 (11.3) | ref | ref |
|  | 2 | 2159 (91.1) | 211 (8.9) | **0.77 (0.64-0.91, p=0.003)** | 0.94 (0.77-1.15, p=0.565) |
|  | >2 | 355 (94.2) | 22 (5.8) | **0.49 (0.30-0.74, p=0.001)** | 0.70 (0.42-1.10, p=0.134) |
|  | missing | 323 (89.5) | 38 (10.5) | 0.92 (0.64-1.30, p=0.663) | 0.98 (0.67-1.39, p=0.900) |
| Subclass | Dapagliflozin | 1126 (91.9) | 99 (8.1) | ref | ref |
|  | Empagliflozin | 4926 (89.4) | 581 (10.6) | **1.34 (1.08-1.68, p=0.010)** | **1.29 (1.03-1.62, p=0.028)** |
| Prior exposure | No | 5796 (89.9) | 651 (10.1) | ref | ref |
|  | Yes | 256 (89.8) | 29 (10.2) | 1.01 (0.67-1.47, p=0.966) | 1.05 (0.69-1.54, p=0.799) |
| Prior antifungal use | No | 5207 (90.1) | 571 (9.9) | ref | ref |
|  | Yes | 845 (88.6) | 109 (11.4) | 1.18 (0.94-1.46, p=0.143) | 1.05 (0.84-1.31, p=0.659) |

**ESM Table 4 Clinical characteristics associated with intolerance versus adherence among SGLT2i users**

|  |  | Adherent | Intolerance | OR (univariable) | OR (multivariable) |  |
| --- | --- | --- | --- | --- | --- | --- |
| Age group | <55 | 1417 (89.6) | 165 (10.4) | ref | ref |  |
|  | 55 to <60 | 1057 (90.7) | 108 (9.3) | 0.88 (0.68-1.13, p=0.316) | 0.86 (0.66-1.11, p=0.252) |  |
|  | 60 to <70 | 2253 (89.6) | 262 (10.4) | 1.00 (0.81-1.23, p=0.990) | 1.01 (0.81-1.26, p=0.936) |  |
|  | ≥70 | 1325 (85.4) | 226 (14.6) | **1.46 (1.18-1.82, p<0.001)** | **1.42 (1.09-1.84, p=0.008)** |  |
| sex | Female | 2270 (87.1) | 336 (12.9) | ref | ref |  |
|  | Male | 3782 (89.9) | 425 (10.1) | **0.76 (0.65-0.88, p<0.001)** | **0.78 (0.67-0.91, p=0.002)** |  |
| Social Deprivation | 1(Most) | 1285 (89.7) | 148 (10.3) | ref | ref |  |
|  | 2 | 1257 (89.7) | 145 (10.3) | 1.00 (0.79-1.28, p=0.990) | 0.99 (0.78-1.27, p=0.958) |  |
|  | 3 | 1233 (88.1) | 166 (11.9) | 1.17 (0.92-1.48, p=0.193) | 1.17 (0.92-1.48, p=0.208) |  |
|  | 4 | 1006 (86.9) | 151 (13.1) | **1.30 (1.02-1.66, p=0.031)** | **1.29 (1.01-1.64, p=0.044)** |  |
|  | 5(Least) | 972 (88.9) | 121 (11.1) | 1.08 (0.84-1.39, p=0.549) | 1.05 (0.81-1.35, p=0.737) |  |
|  | (Missing) | 299 (90.9) | 30 (9.1) | 0.87 (0.57-1.30, p=0.512) | 0.84 (0.54-1.25, p=0.407) |  |
| BMI (kg/m^2^) | <30 | 1427 (86.1) | 231 (13.9) | ref | ref |  |
|  | 30 to <35 | 1751 (90.1) | 193 (9.9) | **0.68 (0.56-0.83, p<0.001)** | **0.70 (0.57-0.87, p=0.001)** |  |
|  | 35 to <40 | 1273 (90.0) | 141 (10.0) | **0.68 (0.55-0.85, p=0.001)** | **0.71 (0.56-0.89, p=0.003)** |  |
|  | ≥40 | 952 (89.8) | 108 (10.2) | **0.70 (0.55-0.89, p=0.004)** | **0.72 (0.55-0.92, p=0.011)** |  |
|  | missing | 649 (88.1) | 88 (11.9) | 0.84 (0.64-1.09, p=0.186) | 0.85 (0.64-1.11, p=0.237) |  |
| HbA_1c_ (mmol/mol) | <70 | 2072 (89.6) | 241 (10.4) | ref | ref |  |
|  | 70 to <80 | 1434 (88.2) | 191 (11.8) | 1.15 (0.94-1.40, p=0.187) | 1.14 (0.93-1.39, p=0.222) |  |
|  | 80 to <90 | 1070 (88.5) | 139 (11.5) | 1.12 (0.89-1.39, p=0.328) | 1.13 (0.90-1.41, p=0.287) |  |
|  | ≥90 | 1073 (88.7) | 137 (11.3) | 1.10 (0.88-1.37, p=0.411) | 1.05 (0.84-1.32, p=0.648) |  |
|  | missing | 403 (88.4) | 53 (11.6) | 1.13 (0.82-1.54, p=0.446) | 1.09 (0.78-1.50, p=0.612) |  |
| CKD stage | 1 | 3215 (89.4) | 381 (10.6) | ref | ref |  |
|  | 2 | 2159 (88.3) | 286 (11.7) | 1.12 (0.95-1.31, p=0.180) | 0.95 (0.78-1.14, p=0.563) |  |
|  | >2 | 355 (87.4) | 51 (12.6) | 1.21 (0.88-1.64, p=0.227) | 0.99 (0.70-1.39, p=0.977) |  |
|  | missing | 323 (88.3) | 43 (11.7) | 1.12 (0.79-1.55, p=0.497) | 1.04 (0.73-1.47, p=0.806) |  |
| Subclass | Dapagliflozin | 1126 (90.4) | 120 (9.6) | ref | ref |  |
|  | Empagliflozin | 4926 (88.5) | 641 (11.5) | 1.22 (1.00-1.51, p=0.057) | **1.25 (1.02-1.55, p=0.036)** |  |
| Prior exposure | No | 5796 (88.9) | 725 (11.1) | ref | ref |  |
|  | Yes | 256 (87.7) | 36 (12.3) | 1.12 (0.77-1.59, p=0.521) | 1.07 (0.73-1.52, p=0.718) |  |
| Prior antifungal use | No | 5207 (89.5) | 608 (10.5) | ref | ref |  |
|  | Yes | 845 (84.7) | 153 (15.3) | **1.55 (1.28-1.87, p<0.001)** | **1.54 (1.26-1.87, p<0.001)** |  |

**ESM Table 5 GLP-1RA baseline characteristics by drug usage category**

|  |  | Adherent | Poor adherence | Discontinued Standard | Discontinued  Upper Bound | Discontinued  Intolerance |
| --- | --- | --- | --- | --- | --- | --- |
| N (%) |  | 2554 (65.7) | 420 (10.8) | 289 (7.4) | 375 (9.7) | 247 (6.4) |
| Subclass | Semaglutide | 406 (15.9) | 35 (8.3) | 40 (13.8) | 51 (13.6) | 31 (12.6) |
|  | Dulaglutide | 628 (24.6) | 67 (16.0) | 53 (18.3) | 70 (18.7) | 57 (23.1) |
|  | Liraglutide | 1047 (41.0) | 239 (56.9) | 111 (38.4) | 109 (29.1) | 106 (42.9) |
|  | Exenatide SR | 261 (10.2) | 37 (8.8) | 42 (14.5) | 62 (16.5) | 22 (8.9) |
|  | Lixisenatide | 67 (2.6) | 20 (4.8) | 17 (5.9) | 32 (8.5) | 12 (4.9) |
|  | Exenatide | 145 (5.7) | 22 (5.2) | 26 (9.0) | 51 (13.6) | 19 (7.7) |
| Prior subclass | Yes | 605 (23.7) | 117 (27.9) | 80 (27.7) | 117 (31.2) | 56 (22.7) |
| Age (years) | Median (IQR) | 61.5 (55.1 - 67.8) | 58.7 (52.9 - 65.6) | 59.2 (51.5 - 66.5) | 62.6 (56.5 - 69.0) | 62.1 (55.2 - 68.8) |
| sex | F | 1167 (45.7) | 187 (44.5) | 139 (48.1) | 167 (44.5) | 137 (55.5) |
| Social Deprivation | 1 (Most) | 573 (22.4) | 117 (27.9) | 84 (29.1) | 89 (23.7) | 65 (26.3) |
|  | 2 | 551 (21.6) | 73 (17.4) | 62 (21.5) | 83 (22.1) | 48 (19.4) |
|  | 3 | 518 (20.3) | 94 (22.4) | 53 (18.3) | 64 (17.1) | 53 (21.5) |
|  | 4 | 422 (16.5) | 63 (15.0) | 45 (15.6) | 72 (19.2) | 31 (12.6) |
|  | 5 (Least) | 378 (14.8) | 53 (12.6) | 31 (10.7) | 54 (14.4) | 35 (14.2) |
|  | (Missing) | 112 (4.4) | 20 (4.8) | 14 (4.8) | 13 (3.5) | 15 (6.1) |
| Diabetes duration | Median (IQR) | 10.2 (6.6 - 14.4) | 9.5 (5.9 - 13.7) | 8.9 (5.0 - 13.1) | 10.4 (5.8 - 15.3) | 9.7 (5.9 - 15.2) |
| Year | 2008-2012 | 670 (26.2) | 147 (35.0) | 84 (29.1) | 104 (27.7) | 68 (27.5) |
|  | 2013-2015 | 431 (16.9) | 90 (21.4) | 67 (23.2) | 84 (22.4) | 49 (19.8) |
|  | 2016-2018 | 529 (20.7) | 77 (18.3) | 50 (17.3) | 70 (18.7) | 49 (19.8) |
|  | 2019-2022 | 924 (36.2) | 106 (25.2) | 88 (30.4) | 117 (31.2) | 81 (32.8) |
| BMI (kg/m²) | Median (IQR) | 36.6 (33.0 - 41.1) | 36.8 (33.4 - 41.6) | 35.7 (32.2 - 41.8) | 36.3 (32.5 - 41.1) | 35.9 (32.2 - 40.0) |
| HbA1c (mmol/mol) | Median (IQR) | 80.0 (70.0 - 92.0) | 80.5 (69.2 - 96.0) | 84.0 (72.8 - 95.0) | 80.0 (71.0 - 93.0) | 80.0 (71.0 - 93.0) |
| eGFR(mL/min/1.73m²) | Median (IQR) | 91.8 (74.5 - 101.5) | 92.5 (77.6 - 104.0) | 92.8(76.3 - 104.2) | 88.0 (69.3 - 99.7) | 88.3 (65.7 - 98.9) |
| Comedications: | Metformin | 2133 (83.5) | 348 (82.9) | 223 (77.2) | 298 (79.5) | 174 (70.4) |
|  | Sulphonylureas | 1344 (52.6) | 211 (50.2) | 135 (46.7) | 183 (48.8) | 98 (39.7) |
|  | TZD | 420 (16.4) | 81 (19.3) | 40 (13.8) | 73 (19.5) | 34 (13.8) |
|  | DPP4-i | 887 (34.7) | 128 (30.5) | 97 (33.6) | 118 (31.5) | 73 (29.6) |
|  | GLP1-RA | 342 (13.4) | 63 (15) | 30 (10.4) | 44 (11.7) | 21 (8.5) |
|  | SGLT2-i | 544 (21.3) | 66 (15.7) | 50 (17.3) | 64 (17.1) | 41 (16.6) |
|  | Insulin | 611 (23.9) | 93 (22.1) | 83 (28.7) | 127 (33.9) | 88 (35.6) |
|  | Antihypertensives | 1883 (73.7) | 310 (73.8) | 191 (66.1) | 287 (76.5) | 167(67.6) |
|  | Statins | 2027 (79.4) | 318 (75.7) | 210 (72.7) | 279 (74.4) | 169(68.4) |

**ESM Table 6 Clinical characteristics associated with poor adherence versus adherence among GLP-1RA users**

|  |  | Adherent | Poor adherence | OR (univariable) | OR (multivariable) |
| --- | --- | --- | --- | --- | --- |
| Age group | <55 | 618 (80.4) | 151 (19.6) | ref | ref |
|  | 55 to <60 | 522 (86.6) | 81 (13.4) | **0.64 (0.47-0.85, p=0.002)** | **0.66 (0.48-0.89, p=0.007)** |
|  | 60 to <70 | 970 (88.3) | 128 (11.7) | **0.54 (0.42-0.70, p<0.001)** | **0.56 (0.42-0.74, p<0.001)** |
|  | ≥70 | 444 (88.1) | 60 (11.9) | **0.55 (0.40-0.76, p<0.001)** | **0.62 (0.42-0.91, p=0.017)** |
| sex | Female | 1167 (86.2) | 187 (13.8) | ref | ref |
|  | Male | 1387 (85.6) | 233 (14.4) | 1.05 (0.85-1.29, p=0.656) | 1.12 (0.90-1.39, p=0.306) |
| Social Deprivation | 1(Most) | 573 (83.0) | 117 (17.0) | ref | ref |
|  | 2 | 551 (88.3) | 73 (11.7) | **0.65 (0.47-0.89, p=0.007)** | **0.65 (0.47-0.90, p=0.009)** |
|  | 3 | 518 (84.6) | 94 (15.4) | 0.89 (0.66-1.19, p=0.435) | 0.92 (0.68-1.25, p=0.612) |
|  | 4 | 422 (87.0) | 63 (13.0) | 0.73 (0.52-1.01, p=0.064) | 0.75 (0.53-1.06, p=0.104) |
|  | 5(Least) | 378 (87.7) | 53 (12.3) | **0.69 (0.48-0.97, p=0.035)** | 0.74 (0.51-1.05, p=0.096) |
|  | (Missing) | 112 (84.8) | 20 (15.2) | 0.87 (0.51-1.44, p=0.610) | 0.92 (0.53-1.53, p=0.744) |
| BMI (kg/m^2^) | <30 | 223 (90.7) | 23 (9.3) | ref | ref |
|  | 30 to <35 | 693 (85.1) | 121 (14.9) | **1.69 (1.08-2.77, p=0.028)** | 1.54 (0.97-2.54, p=0.078) |
|  | 35 to <40 | 764 (86.9) | 115 (13.1) | 1.46 (0.93-2.39, p=0.116) | 1.26 (0.79-2.09, p=0.340) |
|  | ≥40 | 700 (84.1) | 132 (15.9) | **1.83 (1.17-2.99, p=0.011)** | 1.52 (0.95-2.51, p=0.090) |
|  | missing | 174 (85.7) | 29 (14.3) | 1.62 (0.91-2.92, p=0.106) | 1.67 (0.91-3.07, p=0.098) |
| HbA_1c_ (mmol/mol) | <70 | 636 (85.6) | 107 (14.4) | ref | ref |
|  | 70 to <80 | 615 (87.2) | 90 (12.8) | 0.87 (0.64-1.18, p=0.365) | 0.87 (0.64-1.18, p=0.376) |
|  | 80 to <90 | 522 (89.4) | 62 (10.6) | **0.71 (0.50-0.98, p=0.041)** | 0.74 (0.53-1.04, p=0.089) |
|  | ≥90 | 676 (83.4) | 135 (16.6) | 1.19 (0.90-1.57, p=0.223) | 1.26 (0.95-1.68, p=0.108) |
|  | missing | 105 (80.2) | 26 (19.8) | 1.47 (0.90-2.34, p=0.111) | 1.61 (0.95-2.65, p=0.069) |
| CKD stage | 1 | 1316 (85.1) | 230 (14.9) | ref | ref |
|  | 2 | 842 (86.3) | 134 (13.7) | 0.91 (0.72-1.14, p=0.425) | 1.11 (0.85-1.44, p=0.442) |
|  | >2 | 282 (88.1) | 38 (11.9) | 0.77 (0.53-1.10, p=0.164) | 1.04 (0.67-1.57, p=0.856) |
|  | missing | 114 (86.4) | 18 (13.6) | 0.90 (0.52-1.48, p=0.700) | 0.88 (0.49-1.51, p=0.664) |
| Subclass | Semaglutide | 406 (92.1) | 35 (7.9) | ref | ref |
|  | Dulaglutide | 628 (90.4) | 67 (9.6) | 1.24 (0.81-1.92, p=0.328) | 1.24 (0.81-1.93, p=0.331) |
|  | Liraglutide | 1047 (81.4) | 239 (18.6) | **2.65 (1.85-3.90, p<0.001)** | **3.07 (2.11-4.60, p<0.001)** |
|  | Exenatide SR | 261 (87.6) | 37 (12.4) | **1.64 (1.01-2.69, p=0.046)** | 1.46 (0.89-2.41, p=0.137) |
|  | Lixisenatide | 67 (77.0) | 20 (23.0) | **3.46 (1.86-6.31, p<0.001)** | **3.59 (1.90-6.65, p<0.001)** |
|  | Exenatide | 145 (86.8) | 22 (13.2) | 1.76 (0.99-3.08, p=0.050) | **2.21 (1.21-3.96, p=0.009)** |
| Prior exposure | No | 1949 (86.5) | 303 (13.5) | ref | ref |
|  | Yes | 605 (83.8) | 117 (16.2) | 1.24 (0.98-1.56, p=0.065) | **1.66 (1.28-2.15, p<0.001)** |

**ESM Table 7 Clinical characteristics associated with intolerance versus adherence among GLP-1RA users**

|  |  | **Adherent** | **Intolerance** | **OR (univariable)** | **OR (multivariable)** |
| --- | --- | --- | --- | --- | --- |
| Age group | <55 | 618 (91.3) | 59 (8.7) | ref | ref |
|  | 55 to <60 | 522 (92.9) | 40 (7.1) | 0.80 (0.53-1.21, p=0.303) | 0.80 (0.52-1.22, p=0.303) |
|  | 60 to <70 | 970 (91.1) | 95 (8.9) | 1.03 (0.73-1.45, p=0.883) | 0.99 (0.69-1.44, p=0.956) |
|  | ≥70 | 444 (89.3) | 53 (10.7) | 1.25 (0.84-1.85, p=0.262) | 1.09 (0.67-1.75, p=0.737) |
| sex | Female | 1167 (89.5) | 137 (10.5) | ref | ref |
|  | Male | 1387 (92.7) | 110 (7.3) | **0.68 (0.52-0.88, p=0.003)** | **0.66 (0.50-0.86, p=0.002)** |
| Social Deprivation | 1(Most) | 573 (89.8) | 65 (10.2) | ref | ref |
|  | 2 | 551 (92.0) | 48 (8.0) | 0.77 (0.52-1.13, p=0.186) | 0.73 (0.49-1.09, p=0.122) |
|  | 3 | 518 (90.7) | 53 (9.3) | 0.90 (0.61-1.32, p=0.596) | 0.85 (0.57-1.25, p=0.410) |
|  | 4 | 422 (93.2) | 31 (6.8) | 0.65 (0.41-1.00, p=0.056) | **0.62 (0.39-0.97, p=0.040)** |
|  | 5(Least) | 378 (91.5) | 35 (8.5) | 0.82 (0.53-1.25, p=0.356) | 0.78 (0.50-1.20, p=0.257) |
|  | (Missing) | 112 (88.2) | 15 (11.8) | 1.18 (0.63-2.09, p=0.586) | 1.15 (0.61-2.07, p=0.642) |
| BMI (kg/m^2^) | <30 | 223 (87.1) | 33 (12.9) | ref | ref |
|  | 30 to <35 | 693 (92.0) | 60 (8.0) | **0.59 (0.38-0.93, p=0.020)** | **0.59 (0.38-0.94, p=0.024)** |
|  | 35 to <40 | 764 (91.2) | 74 (8.8) | 0.65 (0.43-1.02, p=0.057) | 0.64 (0.41-1.01, p=0.050) |
|  | ≥40 | 700 (92.5) | 57 (7.5) | **0.55 (0.35-0.87, p=0.010)** | **0.51 (0.32-0.82, p=0.005)** |
|  | missing | 174 (88.3) | 23 (11.7) | 0.89 (0.50-1.57, p=0.697) | 0.89 (0.49-1.61, p=0.712) |
| HbA_1c_ (mmol/mol) | <70 | 636 (91.8) | 57 (8.2) | ref | ref |
|  | 70 to <80 | 615 (91.0) | 61 (9.0) | 1.11 (0.76-1.62, p=0.599) | 1.07 (0.73-1.58, p=0.713) |
|  | 80 to <90 | 522 (92.1) | 45 (7.9) | 0.96 (0.64-1.44, p=0.852) | 0.96 (0.63-1.45, p=0.843) |
|  | ≥90 | 676 (90.7) | 69 (9.3) | 1.14 (0.79-1.65, p=0.487) | 1.11 (0.77-1.63, p=0.572) |
|  | missing | 105 (87.5) | 15 (12.5) | 1.59 (0.84-2.85, p=0.131) | 1.45 (0.74-2.70, p=0.252) |
| CKD stage | 1 | 1316 (92.2) | 111 (7.8) | ref | ref |
|  | 2 | 842 (91.4) | 79 (8.6) | 1.11 (0.82-1.50, p=0.488) | 1.09 (0.78-1.53, p=0.608) |
|  | >2 | 282 (87.0) | 42 (13.0) | **1.77 (1.20-2.56, p=0.003)** | **1.65 (1.04-2.58, p=0.029)** |
|  | missing | 114 (88.4) | 15 (11.6) | 1.56 (0.85-2.69, p=0.128) | 1.40 (0.73-2.50, p=0.284) |
| Subclass | Semaglutide | 406 (92.9) | 31 (7.1) | ref | ref |
|  | Dulaglutide | 628 (91.7) | 57 (8.3) | 1.19 (0.76-1.89, p=0.456) | 1.16 (0.73-1.85, p=0.536) |
|  | Liraglutide | 1047 (90.8) | 106 (9.2) | 1.33 (0.89-2.04, p=0.184) | 1.50 (0.98-2.35, p=0.068) |
|  | Exenatide SR | 261 (92.2) | 22 (7.8) | 1.10 (0.62-1.94, p=0.733) | 1.13 (0.63-2.00, p=0.683) |
|  | Lixisenatide | 67 (84.8) | 12 (15.2) | **2.35 (1.11-4.69, p=0.019)** | **2.28 (1.07-4.62, p=0.026)** |
|  | Exenatide | 145 (88.4) | 19 (11.6) | 1.72 (0.93-3.11, p=0.079) | 1.82 (0.95-3.39, p=0.064) |
| Prior exposure | No | 1949 (91.1) | 191 (8.9) | ref | ref |
|  | Yes | 605 (91.5) | 56 (8.5) | 0.94 (0.69-1.28, p=0.719) | 0.99 (0.71-1.38, p=0.975) |
